# Supplementary material for: Plasmonic Brownian Ratchets for Directed Transport of Analytes
Source: Nano Lett. 2025 Nov 26;26(1):181–7. doi: 10.1021/acs.nanolett.5c04804 (PMC12810488; doi:10.1021/acs.nanolett.5c04804)
Supplement: Supplementary file 1 [file nl5c04804_si_001.pdf]

Supporting Information to Plasmonic brownian ratchets for directed transport of analytes

Marciano Palma do Carmo,*,† David Mack,‡ Diane J. Roth,† Miao Zhao,† Ancin M. Devis,† Francisco J. Rodríguez-Fortuño,† Stefan A. Maier,¶,‡ Paloma A. Huidobro,§,‖ and Aliaksandra Rakovich*,†

†*Physics Department, King's College London, London WC2R 2LS, UK*

‡*Department of Physics, Imperial College London, London SW7 2AZ, UK*

¶*School of Physics and Astronomy, Monash Unversity, Clayton Victoria 316, Australia*

§*Departamento de Física Teórica de la Materia Condensada, Universidad Autónoma de Madrid, 28049 Madrid, Spain*

‖*Condensed Matter Physics Center (IFIMAC), Universidad Autónoma de Madrid, 28049 Madrid, Spain*

E-mail: marciano.palma_do_carmo@kcl.ac.uk; aliaksandra.rakovic@kcl.ac.uk

Principles of Brownian Ratchets

The working principle of a Brownian Ratchet involves the periodic trapping of analytes by an asymmetric potential and the free diffusion of the analytes between the periods of trapping, as depicted schematically in Figure S1. The key requirements for rectification of Brownian motion to occur is that the potential (ΔU) remains sufficiently deep to trap the analytes and

that the time that said potential remains off (τ_{off}) lies between the two limits corresponding to the time it takes for analyte particles to diffuse forward (τ_F) and backwards (τ_B) to the adjacent potential unit cells from the position of the potential minima, *i.e.* distances r and $L - r$ in Figure S1, where L is the ratchet period and r is the shortest distance from the potential minimum to the next unit cell. For a 2-dimensional ratchet, with a repeating structure along two different directions and particles diffusing above it, these respective times can be calculated using the diffusion theory:

$$\tau_F = \frac{r^2}{2D} \quad \text{and} \quad \tau_B = \frac{(L-r)^2}{2D}, \tag{S1}$$

where D is the diffusion constant of the analyte particles in solution. The diffusion constant can be estimated from the Stokes–Einstein relation,

$$D = \frac{k_B T}{6\pi\eta R_h}, \tag{S2}$$

where η is the viscosity of the solvent, T the absolute temperature, and R_h the hydrodynamic radius of the analyte particle. For example, for 40 nm PS spheres in water at room temperature ($T = 298$ K, $\eta = 10^{-3}$ Pa·s), $R_h \approx 20$ nm gives $D \approx 10.9$ μm^{2}s^{-1}, which corresponds to $\tau_F \approx 4.6$ ms and $\tau_B \approx 6.7$ ms for the optimized ratchet geometry discussed in the main text.

Using these definitions, the requirement for rectification of Brownian motion by the ratchet can be stated mathematically as:

$$\tau_B > \tau_{off} > \tau_F. \tag{S3}$$

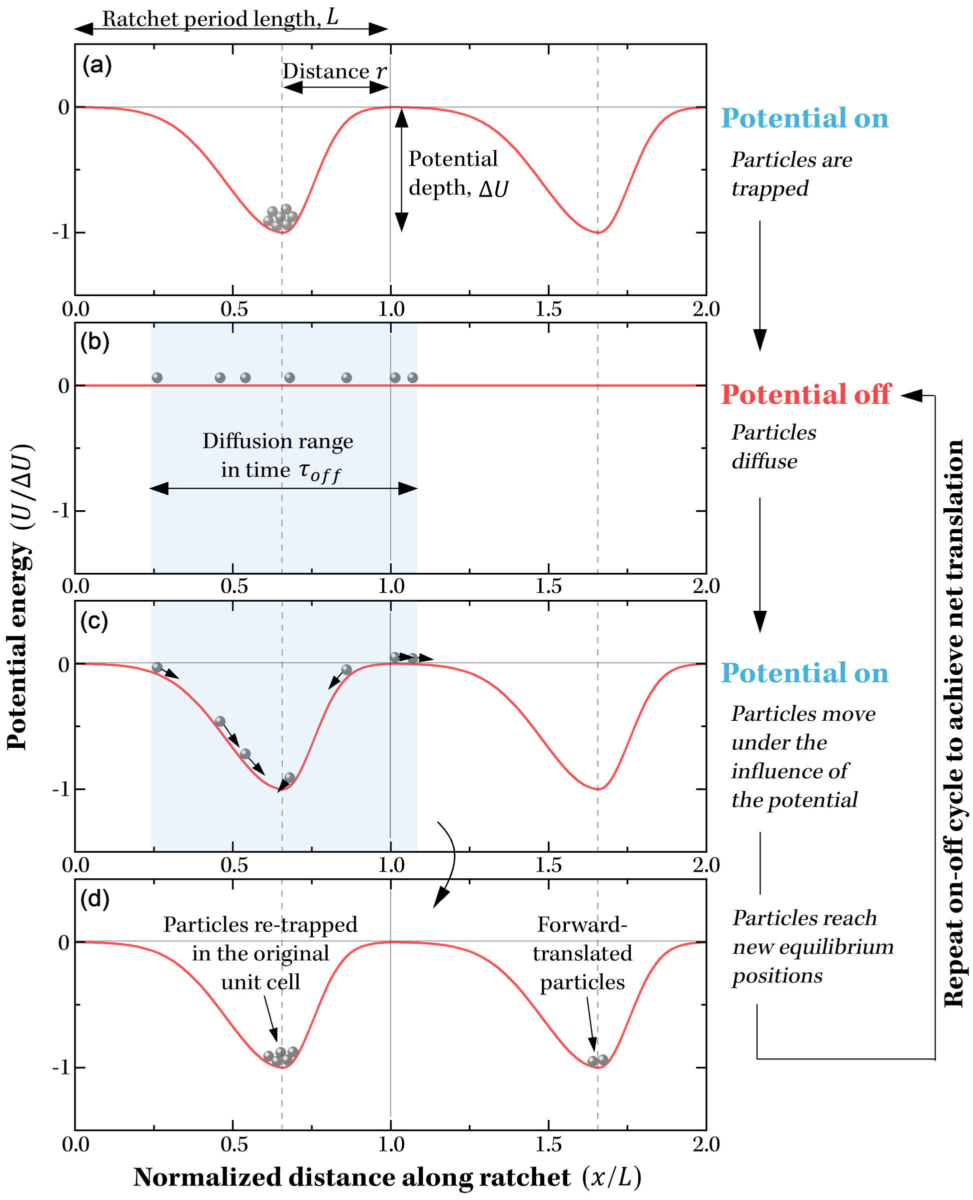

Figure S1: The working principles of a Brownian ratchet. During the times that the trapping potential remains on, τ_{on}, the analyte particles are driven towards locations corresponding to potential minima (panel (c)) and remain trapped there (panels (a) and (d)). When the potential is then turned off (panel (b)), for a period of τ_{off}, the particles are able to diffuse freely in any direction with a characteristic diffusion coefficient, D. Once the potential is turned on again (panel (c)), the particles are re-trapped and driven towards potential minima again (panel (d)). For rectification of Brownian motion to occur, the time that the potential remains off must be larger than the average time it takes for an analyte particle to diffuse towards the next potential well (distance r in the figure) but smaller than the time it would take it to diffuse to the previous potential (i.e. distance $L - r$).

Numerical Simulations

Optimization of ratchet asymmetry via dipole approximation

Numerical simulations of the optical response of ratchet structures under plane wave 980 nm illumination were performed using the RF module of COMSOL Multiphysics. Simulations on individual (single unit cell) structures were performed using a large domain size, with Perfectly Matched Layers (PMLs) as its boundaries; this was done to avoid any reflections from the domain boundaries contributing to results of the simulations. The refractive index of the quartz substrate was taken to be 1.5, and the refractive indices of gold (Au) and chromium (Cr) at the excitation wavelength were taken from references 1 and 2, respectively. Using the results of the simulations, the scattering (σ_{sca}) and absorption (σ_{abs}) cross-sections of the arrays were calculated by normalizing the total intensities scattered and absorbed by the plasmonic structures by that of the incident plane wave.

Structures with asymmetry degrees varying from 0° to 21° were considered, in 3° steps, with the asymmetry degree being defined as the angle θ in Figure 2(a) of the main text. From the simulated electric fields, examples of which are shown in Figure S2(b), optical forces offered by each structure were calculated using the dipole approximation,[3] which assumes that diffusing particles can be approximated to be small dipoles of dimensions much smaller than the excitation wavelength and with homogeneous fields inside of them:

$$\vec{F}(\vec{r}) = \frac{\Re\big[\alpha(\omega)\big]}{4}\,\nabla\vec{E}(\vec{r}) \tag{S4}$$

with $\alpha(\omega)$ being the particle's polarizability, defined as:

$$\alpha(\omega) = 4\pi r^3 \frac{\varepsilon_m(\omega) - \varepsilon_p(\omega)}{\varepsilon_m(\omega) + 2\varepsilon_p(\omega)}, \tag{S5}$$

where ε_m and ε_d are the relative permittivities of the medium and of the particle respectively,

r is the particle radius and $\nabla E(r)$ is the gradient of the electric field. The potential energy of the particle at a specific position above the ratchet, $\vec{r}$, relative to a reference position, $\vec{r}_{ref}$, could then be determined by integrating the optical forces acting on the particle along a straight line between the two points:

$$U(\vec{r}) = \int_{\vec{r}_{ref}}^{\vec{r}} \vec{F}(\vec{r'}) \cdot d\vec{r'} \tag{S6}$$

Typically, the reference position was taken to be the geometrical centre of the space between two adjacent ratchet units.

Trapping potentials above single ratchet unit structures were calculated 10 nm above the top surface of the plasmonic structure. Results of these calculations are shown in Figure S2. Based on these results, asymmetry degree of $\sim 9°$ was deemed to be optimum, as it provided a deep, extended and most asymmetric potential.

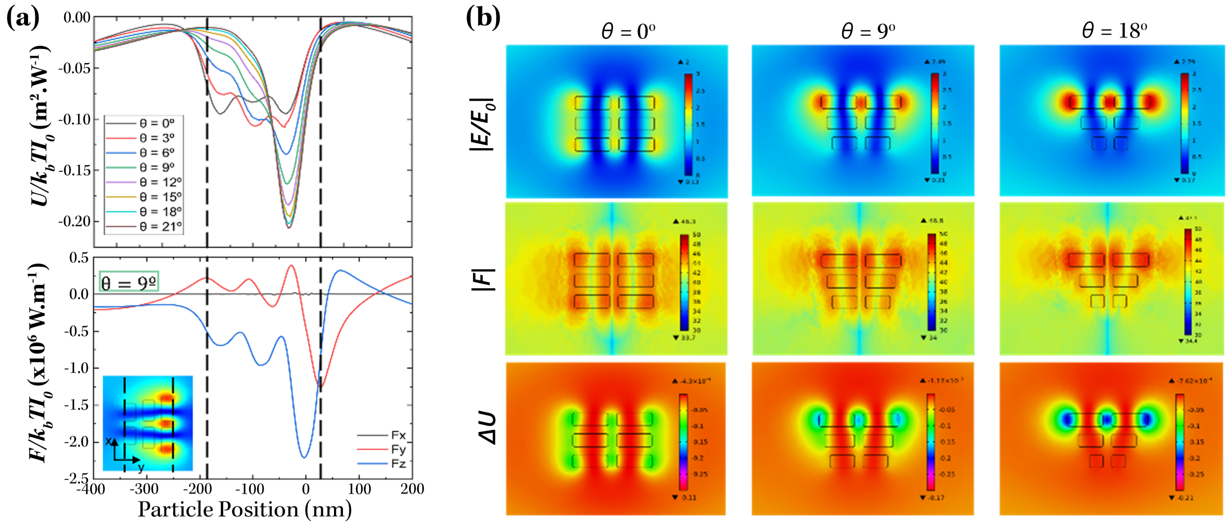

Figure S2: Optimization of ratcheting structure unit cell asymmetry, under dipolar approximation. Top graph of panel (a) shows the trapping potentials for different structure asymmetry degrees θ. Bottom graph of panel (a) shows the x, y and z components of the optical forces generated by the ratcheting unit cell structure of optimum asymmetry degree ($\theta = 9°$). Panel (b) shows electric fields, optical forces and trapping potential profiles for a few select asymmetry degrees of plasmonic ratchet unit cell structures.

Maxwell Stress Tensor calculations of optical forces and potentials

In the study involving optimization of the periodicity of the ratchet (described in the main text), as well as the theoretical predictions of ratchet behavior for nanoparticles of different sizes and compositions, the Maxwell Stress Tensor (MST) method was used to fully account for the effect that the polarization of analyte particle had on the electric fields in its vicinity. To perform these calculations, a set of numerical simulations of the optical response of the ratchet to normally-incident plane wave of 980 nm were performed, with a single dielectric sphere placed at different positions along the central axis and 10 nm above the surface of the ratchet (measured from the top of the ratchet surface to the bottom of the sphere). Periodic boundary conditions were used to simulate an infinite array of the system. For the dielectric sphere, material properties used were those corresponding to either the polystyrene or the PTB7 polymer, as appropriate. The position of the sphere was changed between each simulation, along the ratcheting axis (y direction, see Figure S3), to yield y-distance dependent series of results.

For each simulation in the series, i.e. for each position of the sphere above the ratchet, MST was calculated using:[3,4]

$$\overleftrightarrow{T}(\vec{r},t) = \left[\varepsilon_0\varepsilon\vec{E}\vec{E} + \mu_0\mu\vec{H}\vec{H} - \frac{1}{2}\left(\varepsilon_0\varepsilon E^2 + \mu_0\mu H^2\overleftrightarrow{I}\right)\right] \tag{S7}$$

where ε and μ are the permittivity and permeability of the surrounding medium.

The optical force exerted on a sphere was then calculated by integrating MST over an arbitrary surface δV enclosing the sphere:

$$\vec{F}(\vec{r}) = \int_{\delta V} \langle\overleftrightarrow{T}(\vec{r},t)\rangle\ \hat{n}(\vec{r})\ da \tag{S8}$$

where $\hat{n}(\vec{r})$ is the unit vector normal to the surface at position $\vec{r}$ and pointing outwards from the enclosed volume. Integration was performed over a cubic surface, centered at the

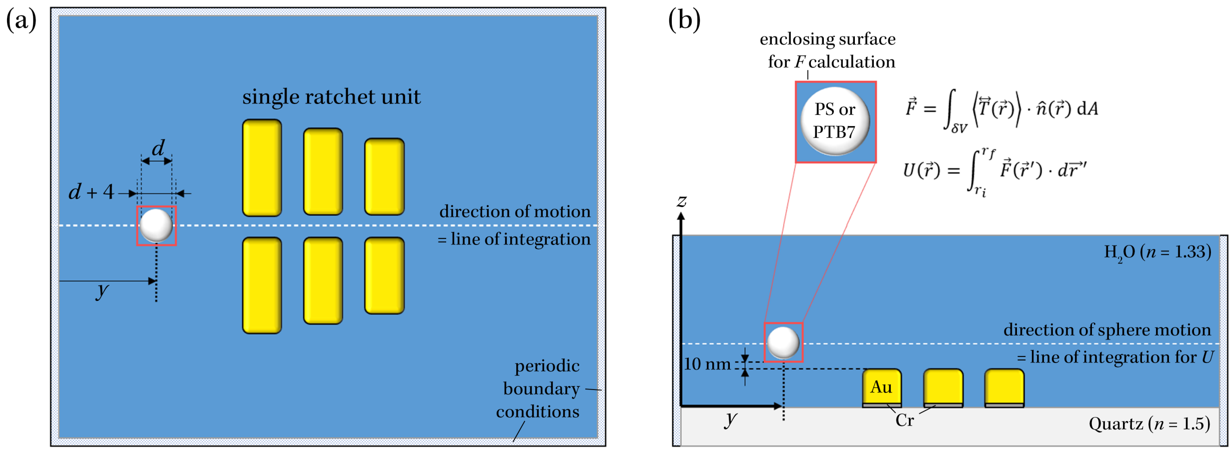

Figure S3: Simulation space set-up for calculations of optical forces and potentials using the Maxwell Stress Tensor, showing the system (a) from the top and (b) from the side. Calculation of trapping potential involved a series of simulations, with varying positions y of the analyte sphere of diameter d, along the ratchet axis (white dashed line) and a height of 10 nm above the top surface of the ratchet. Maxwell Stress Tensor was calculated from the fields yielded by the simulations and then integrated over the surface enclosing the sphere (red solid box), of side length $d + 4$ nm. In all simulations, ratchets were on a quartz substrate and in water. The dimensions and design of the ratchet is described in the main text. Periodic boundary conditions were used to simulate an infinite array of plasmonic ratchets.

dielectric sphere's center and of side length 4 nm larger than the sphere's diameter.

These calculations yielded a set of (F_x, F_y, F_z) as a function of sphere position above and along the central axis of the ratchet, y. The trapping potential along this direction could then be calculated *via* integration according to Equation S6, with the lower limit of integration of $y = 0$ corresponding to the mid-point between two adjacent cells in this case (due to the periodic nature of the structure).

Optimization of ratchet periodicity

Using methods described above, the periodicity of the ratchet array was optimized to yield the deepest trapping potentials. This was achieved by first performing a series of simulations where the yz boundaries of the simulation space had PMLs applied to them, whilst periodic boundary conditions were applied to the xz boundaries, thus simulating a chain of ratcheting

structures, repeated along y direction. The spacing between the ratchets in the chain was controlled by the size of the simulation domain along the y direction, and this was varied between 400 and 1000 nm. These variations caused very strong changes in the optical response of the ratchet, with periodicity of 700 nm for the y-direction offering by far the strongest electric field enhancements, optical forces and trapping potentials (Figure S4).

To determine the optimum periodicity in the x-direction, periodic boundary conditions were additionally applied to the yz boundaries, and the length of the domain along the x direction was varied between 750 and 1000 nm. Variations in the electric fields, optical forces and potentials were very subtle in this case (Figure S5); nonetheless, x-period of 800 nm performed marginally better than other periodicity and was therefore deemed optimum.

Results of simulations for larger nanoparticles

Optical forces and trapping potentials experienced by 200 nm PS spheres and 190 nm PTB7 nanoparticles were calculated using methodology described in above of this supplementary document. Material properties used for these simulations/calculations were taken from 5 and 6, respectively. For the diameters of the dielectric spheres, 200 nm was used for the PS spheres, as this was the value specified by the manufacturer. For PTB7 nanoparticles, 190 nm was used as the diameter; this value was determined experimentally (see Section 2.2). Results of these calculations are shown in Figure S6.

Fabrication protocols

Fabrication of plasmonic ratchets

Arrays of plasmonic structures were fabricated using standard e-beam lithography methods, using 180 nm of 950K A4 Poly(MethylMethacrylate) (MicroChem) as the resist, pre-baked at 180°C for 3 minutes. E-beam write was performed on on e-LINE Plus (Raith) EBL using 20 kV accelerating voltage, 10 μm aperture and 10 μm working distance. Post-write, the

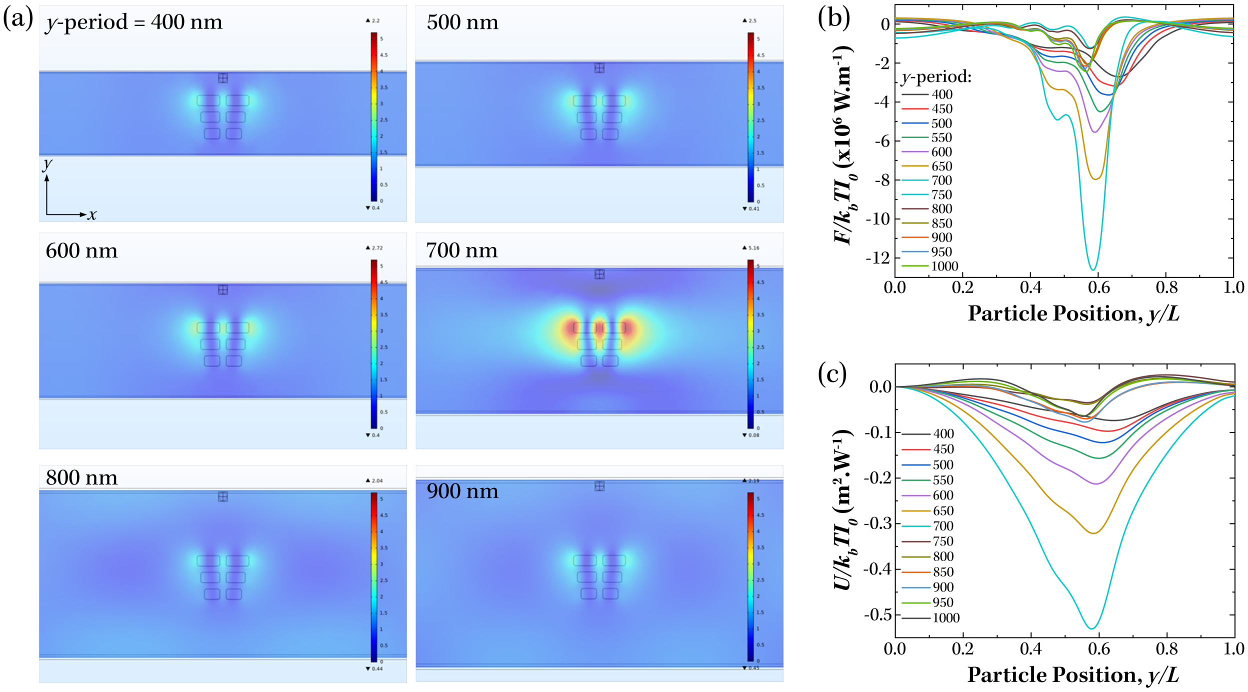

Figure S4: Optimization of y-period of ratchet array using numerical simulations involving a chain of ratchet unit cells, with the period of the unit cell varying between 400 and 1000 nm. (a) Electric field enhancements (E/E_0) above ratchet structures, in the presence of 40 nm polystyrene sphere located 10 nm above the ratchet surface. The profiles are taken at a height cutting through the middle of the sphere ($z = 80$ nm) above the substrate. All profiles are plotted using the same colorscale of $\log_{10}(E/E_0) = 0$ to 5. (b) and (c) show z-component of the optical force and potentials experienced by the polystyrene sphere as a function of its position y along the central axis of the ratchet. In panels (b) and (c), the particle position is plotted in relative units of its displacement from the mid-point of two ratchets to the total length of the ratchet unit cell, L.

resist was developed for 30 seconds in 1:3 methyl isobutyl ketone:isopropyl alcohol (IPA), followed by a further 30 seconds wash in pure IPA. Following development, 5 nm of Cr and 45 nm of Au were evaporated on top of the resist, at a rate of 0.2 Å s^{-1} and 2 Å s^{-1} respectively, using the Amod System evaporator (Angstrom Engineering). Finally, lift-off was performed by immersing the substrate into acetone for 24 hours, followed by rinsing with IPA and deionised water.

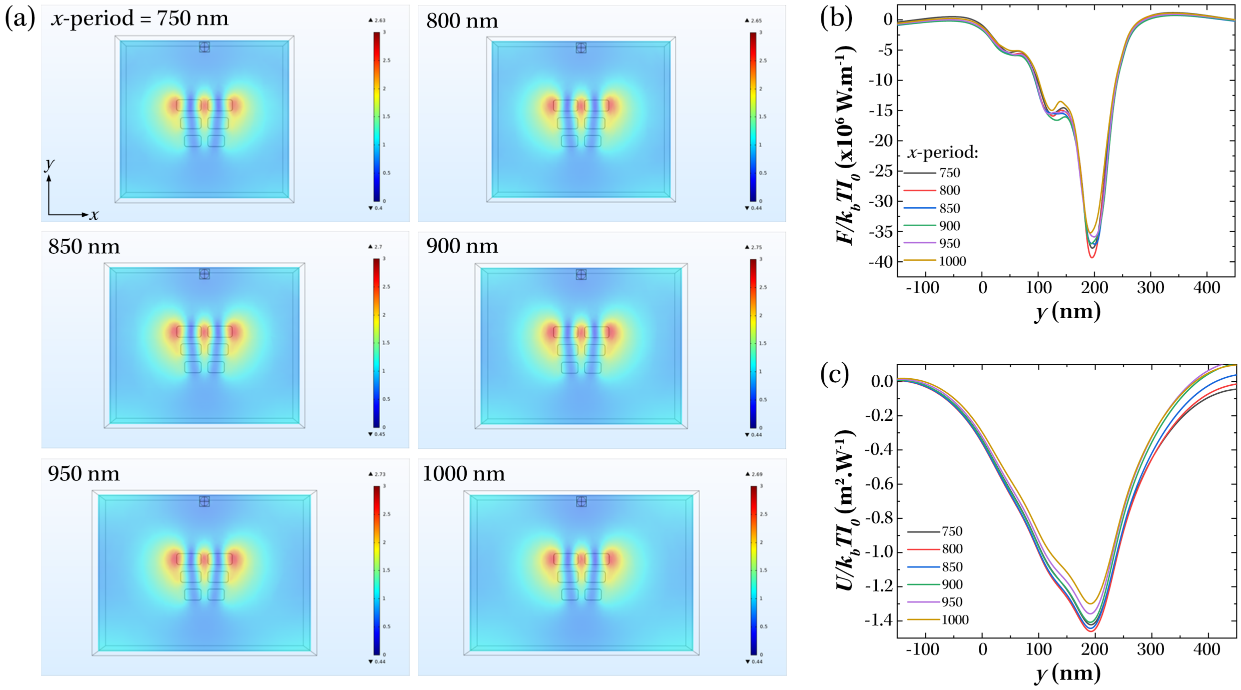

Figure S5: Optimization of x-period of ratchet array using numerical simulations involving an array of ratchets with the y-period set to 700 nm and x-period varied between 750 and 1000 nm. (a) Electric field enhancements (E/E_0) above ratchet structures, in the presence of 40 nm polystyrene sphere located 10 nm above the ratchet surface. The profiles are taken at a height cutting through the middle of the sphere (z = 80 nm) above the substrate. All profiles are plotted using the same colorscale of $\log_{10}(E/E_0) = 0$ to 3. (b) and (c) show z-component of the optical force and potentials experienced by the polystyrene sphere as a function of its position y along the central axis of the ratchet.

Fabrication of PTB7 nanoparticles

A colloidal suspension of PTB7 nanoparticles in water, stabilized by pluronic F127 copolymer, were fabricated as follows. Stock PTB7 and F127 solutions were prepared by dissolving 20 mg of PTB7 and 2 g of F127 in 20 mL of THF each. 10 mL of PTB7 stock solution was then mixed with 10 mL of F127 stock solution, and the mixture was added into 100 mL deionised water whilst being sonicated, for 5 minutes. A stirrer bar was added to the solution and the suspension was left stirring for 3 days. The solution was then topped up with deionised water to 100 mL and left for 2 more days to allow THF to evaporate fully.

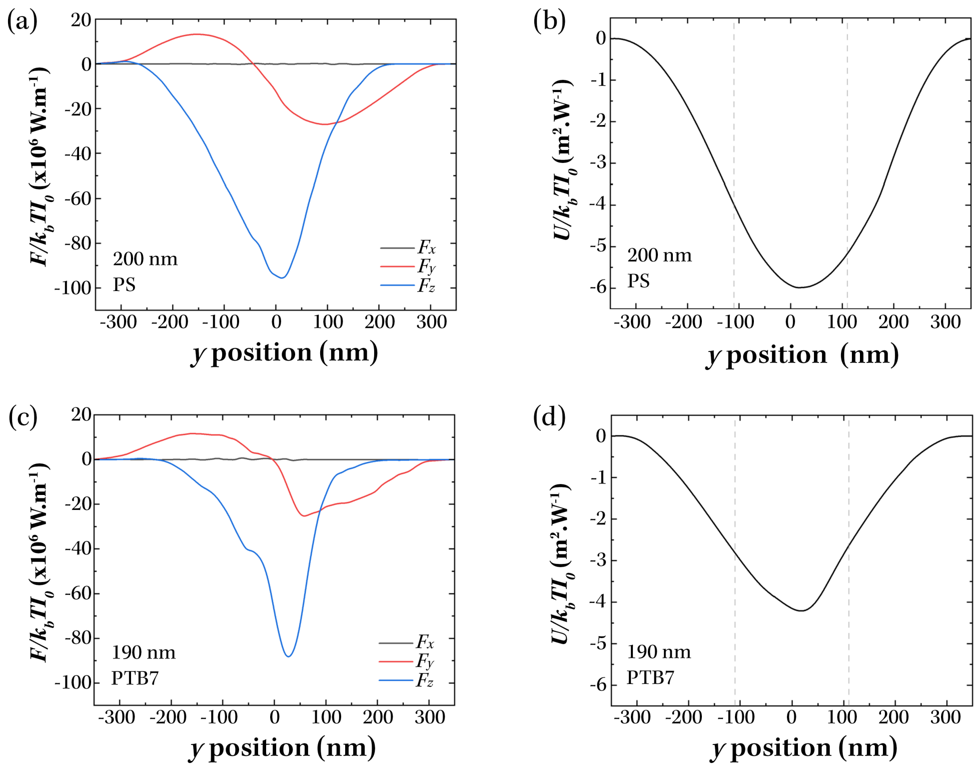

Figure S6: Numerically-calculated optical forces, (a) and (c), and trapping potentials, (b) and (d), experienced by 200 nm PS and PTB7 nanoparticles above the ratchet structure, respectively. The dashed lines in (b) and (d) denote the top and bottom edges of the ratchet structures, respectively.

Particle tracking

Substrates containing plasmonic ratchets were placed into the sample holder fitted to a Nikon Ti-U2 microscope. A small drop of a dielectric particle dispersion (PS or PTB7 beads, 1:10 v/v in water) was placed directly on top of the ratchet array. Sample was illuminated using side-illumination from a white-light source (OSL2, Thorlabs) and light scattered by the dielectric spheres was collected from the other side of the sample using a 40x objective (Nikon, CFI Super Fluor). The scattered light was recorded using KURO sCMOS camera (Princeton Instruments).

To trap the particles, sample was illuminated with a collimated 980 nm CW laser radiation (1.4–3.7 mW, MDL-III-980High Stability Infrared Laser, CNI Laser), incident onto the sample from the top. A 850 nm short-pass filter (ThorLabs) was used to exclude this radia-

tion from recorded images. Chopping of the trapping laser was performed using MC2000B optical chopper (ThorLabs), fitted with a 50%-duty blade.

Videos of at least 3500 frames recorded by the camera were exported as .tiff files and analysed using an own code. Briefly, a video of a specific measurement was imported and the first frame from the video was used to set the xy axis for the analysis, to account for any rotation of the sample in the sample holder. This was done by showing the first frame to the user, who manually drew a line on the image such that it aligned with the edge of the ratchet array. Each frame of the video was then pre-filtered using either a band-pass filter (Python) or using morpholigical operations (Matlab), in order to eliminate extremely small features (*i.e.* noise) and remove background. For each frame, positions and sizes of each particle were identified (in the new reference frame) by detecting all maxima in the image and iteratively applying a circular mask to the image (Python) or by applying blob analysis (Matlab). Results were filtered by applying morphological opperations to exclude particles with sizes above and brightness below a certain threshold, corresponding to removal of data from aggregates of nanoparticles. The positions of particles were then linked from frame to frame, by generating pair-wise associations of particle positions that resulted in a minimum square displacement of all particles from frame to frame. For each identified particle, the code yielded tracks in the form of (x, y, t) data, some of which are shown in Figure 4(d) of main text. A typical analysis yielded 1000 - 1500 tracks per video.

To generate displacement versus time box plots shown in Figure 5(b) and (c) of main text, for each time point on the graph, tracks were first filtered to remove those of insufficient time length (cut-off of 45 ms was used, resulting in N = 80 - 120 remaining trajectories per dataset). For the remainder, tracks were cropped to the cut-off time ($t = 45ms$), and net displacement for each particle was calculated by subtracting its initial position from the final position. The resulting set of displacements was saved, and the process was repeated for other time points. The saved data were then analysed statistically and plotted as box plots using OriginLab. The net displacement versus time data was fitted to a linear model to yield the

mean velocity of the particles in x and y directions. This analysis was repeated for videos obtained at different settings (e.g. different chopping frequencies or different illumination intensities), to yield the corresponding mean velocity versus τ_{off} or intensity graphs shown in Figure 5 of main text.

To evaluate the stiffness of the traps offered by the plasmonic ratchet structure, motion of 40 nm PS particles was recorded at different 980nm laser intensities. Videos were evaluated as above to obtain the net displacement ($\sigma_{x,y}$) of particles in x and y directions over a 45 s time period. The data was then used to calculate the trap stiffness of the ratchet structure in the corresponding directions ($k_{x,y}$) according to $k_{x,y} = k_b T/\sigma^2_{x,y}$.[7,8]

Additional ratcheting data

Ratcheting of 200 nm PS spheres

Experimental observation of ratcheting of 200 nm PS spheres and the analysis of results was performed in a manner identical to those for 40 nm PS spheres; the details of these investigations are described in the main text. Figure S7 shows the results for 200 nm PS spheres.

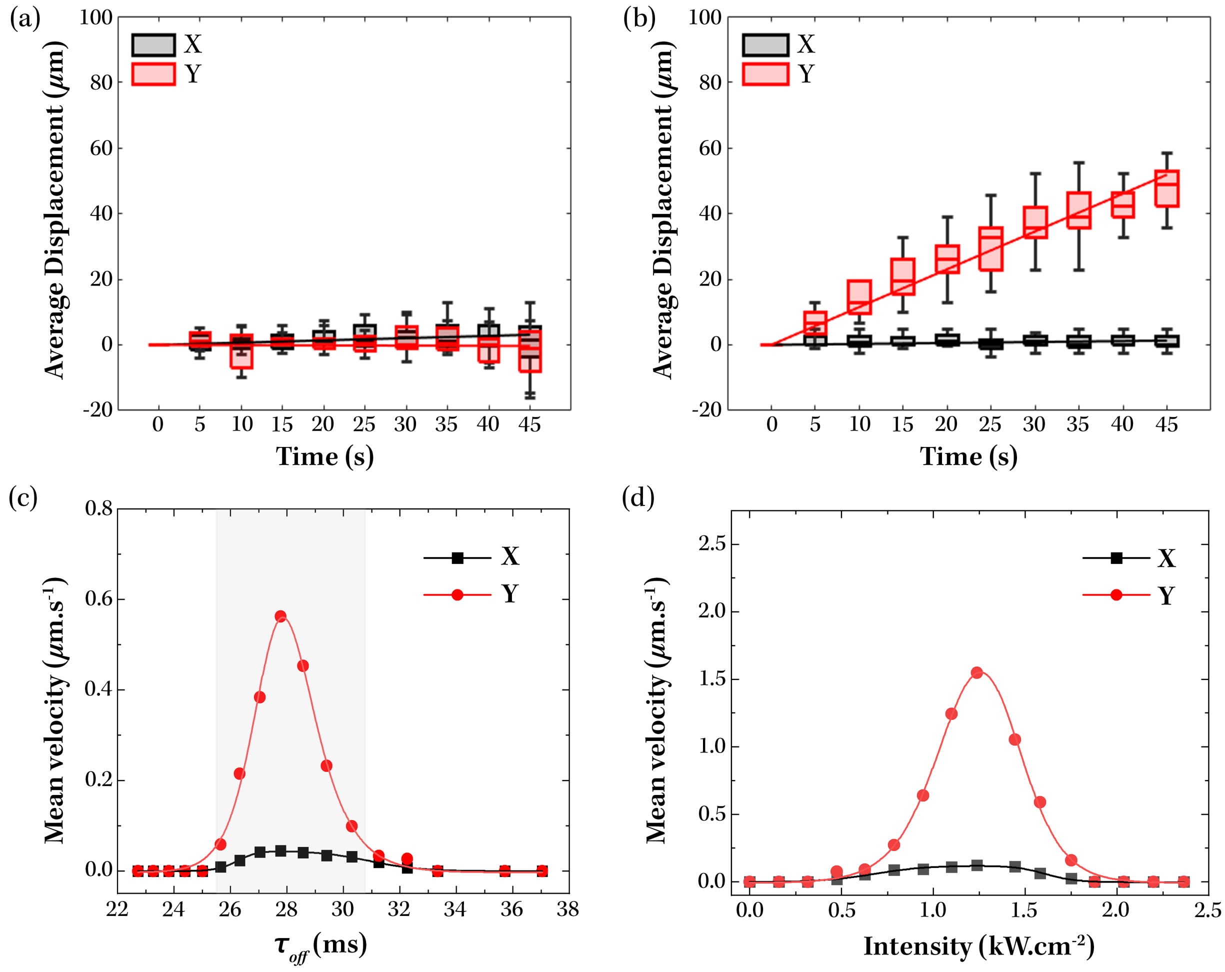

Figure S7: Experimental results for 200 nm PS spheres. Panels (a) and (b) show the average displacement of detected PS spheres as a function of time, for (a) brownian motion and (b) ratcheting motion, respectively. Motion along the y axis corresponded to motion along the ratcheting axis. Results in (a) were obtained with no external 980 nm illumination, whereas those in (b) were obtained with a chopped 980 nm illumination driving the ratchet. Panels (c) and (d) show the mean velocities of 200 nm PS particles along the x and y directions, recorded under chopped 980 nm illumination of ratchets. In (c), the time that the optical potential remained off (τ_{off}) was varied, with the intensity of 980 nm illumination kept constant at $\sim$ 0.67 kW.cm-2. In (d), the intensity of the 980 nm illumination was varied, whist τ_{off} was kept constant at its optimum value of 27.8 ms. The shaded region in (c) represents the theoretically-predicted range of τ_{off} values where ratchetting should be possible.

Ratcheting of PTB7 nanoparticles

Determination of mean diameter of PTB7 nanoparticles

The hydrodynamic diameters of PTB7 particles were measured using a ZetaSizer Nano ZS (Malvern Panalytical). For measurements, 2 mL of 5 μg/mL of PTB7 dispersion was placed into disposable polystyrene cuvettes (DTS0012). Three measurements, with 10 repeats each, were performed at 20°C, with the Malvern software automatically setting the duration of each repeat. Both the correlation curves and the intensity *versus* size curves were indicative of low polydispersity samples (Figure S8). The three measurement results, obtained from cumulative analysis of the correlation curves (performed automatically by Malvern software), were averaged to obtain an average diameter of 203 ± 0.8 nm for PTB7 nanoparticles. Since hydrodynamic radius of nanoparticles is larger than their physical diameter, a somewhat smaller size of 190 nm was used in the theoretical calculations of the optimal range of τ_{off} for these conjugated polymer nanospheres.

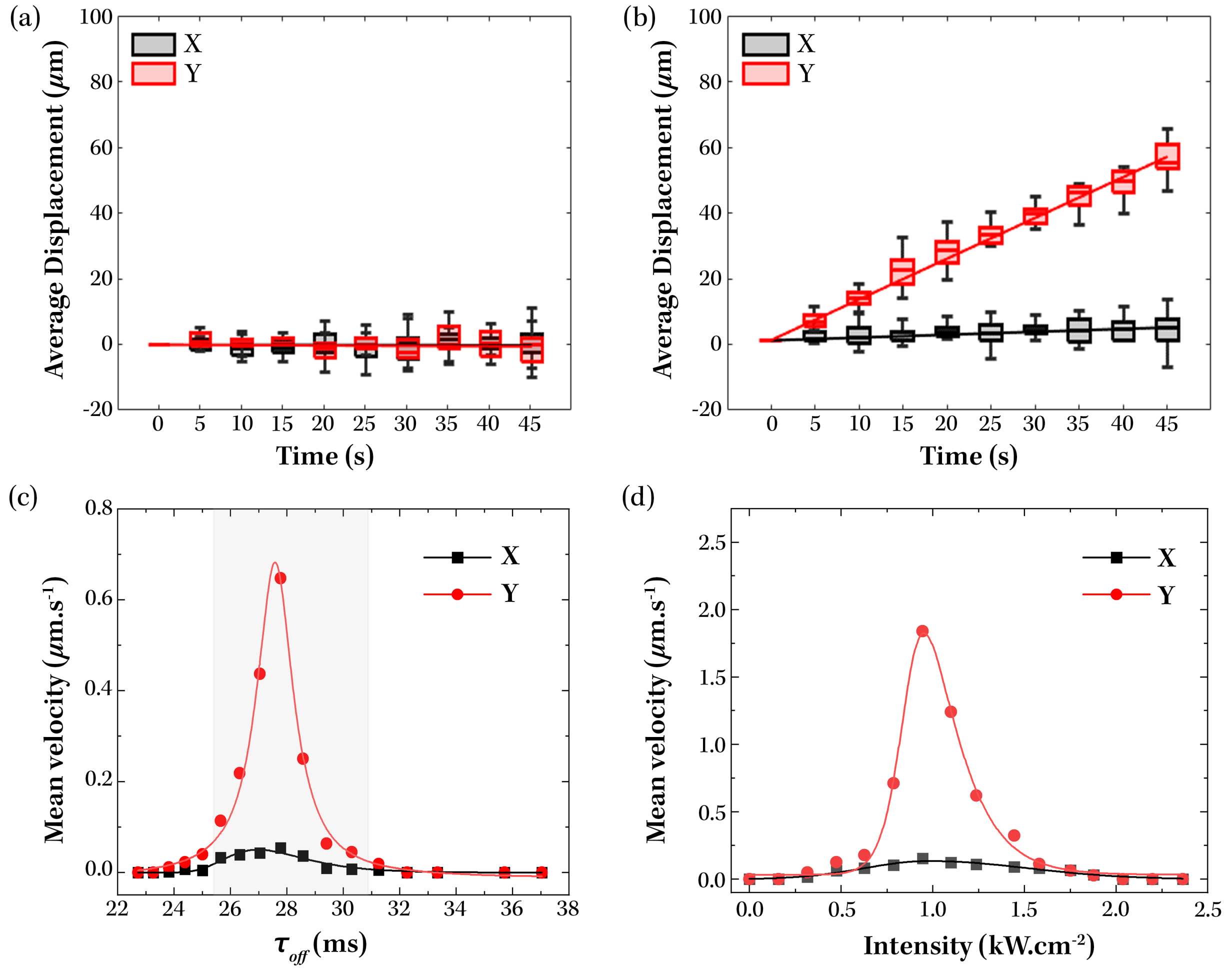

Figure S8: Dynamic Light Scattering measurements of PTB7 nanoparticles in water. Panel (a) shows correlation curves for the three measurements, and panel (b) shows percent of scattered light as a function of particle size. Each measurement consisted of 10 repeats of duration determined automatically by the ZetaSizer Nano ZS software.

Results of ratcheting for PTB7 nanoparticles

Experimental observation of ratcheting of 190 nm PTB7 nanoparticles and the analysis of results was performed in a manner identical to those for 40 nm PS spheres; the details of these investigations are described in the main text. Figure S9 shows the results for 190 nm PTB7 nanoparticles.

Discussion of the decrease in ratcheting efficiency at high intensities

At excitation intensities above approximately 0.8 kW cm^{-2}, the ratcheting efficiency of 40 nm PS nanoparticles began to decrease and eventually vanished beyond 1.5 kW cm^{-2} (Figure 5(b) of the main text). Several mechanisms could contribute to this behavior, including local photothermal heating, thermophoresis, microbubble formation, or changes in the optical force landscape.

Local heating around plasmonic structures is an unavoidable consequence of Ohmic losses, and even modest temperature rises can alter the surrounding fluid environment. In water, heating reduces viscosity by roughly $2-3\%$ K^{-1}, which slightly enhances Brownian diffusivity of the PS spheres, while at the same time generating temperature gradients that can drive thermophoretic motion. For uncharged PS particles in deionized water used in our studies, the direction of this motion would typically be thermophobic, leading to a depletion of spheres near hot regions; this has been previously reported for various plasmonic trapping systems.[9–12] In the present system, bulk temperature measurements under continuous 980 nm illumination revealed an increase below 1° C even at the highest experimental powers used (2.4 kW cm^{-2}), and no visible depletion of particles or fluctuations indicative of fluid flow were observed. On the other hand, and as shown in Figure 5(c) of the main text, the trap stiffness increased rapidly with power, indicating that optical gradient forces remained dominant over any thermophoretic repulsion that was present. Microbubble formation and convective motion can also occur when local temperature rises exceed approximately 80 - 100 K,[10,13] but there was no experimental evidence that such effects occurred here: dur-

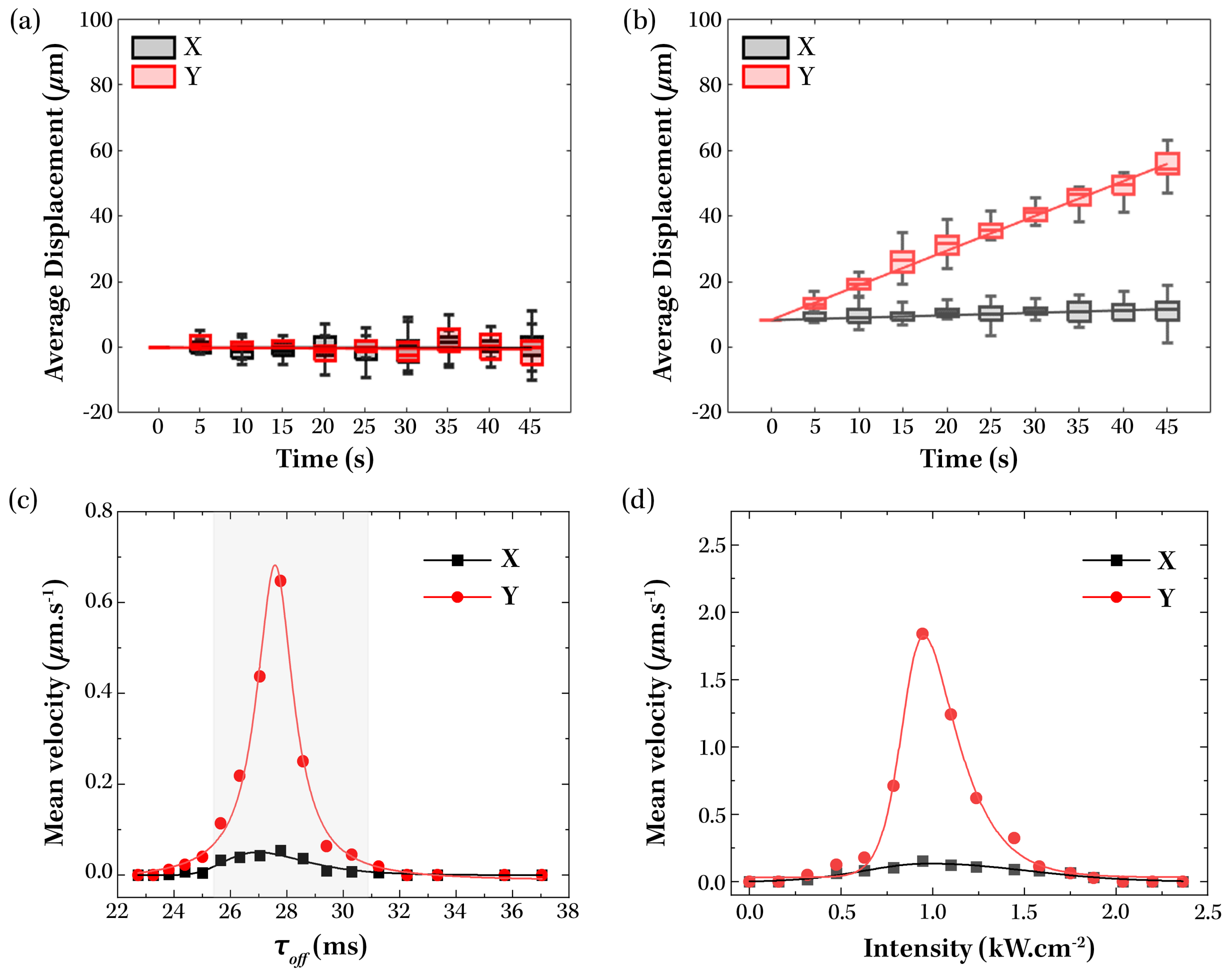

Figure S9: Experimental results for 190 nm PTB7 spheres. Panels (a) and (b) show the average displacement of detected PTB7 nanoparticles as a function of time, for (a) Brownian motion and (b) ratcheting motion, respectively. Motion along the y axis corresponded to motion along the ratcheting axis. Results in (a) were obtained with no external 980 nm illumination, whereas those in (b) were obtained with a chopped 980 nm illumination driving the ratchet. Panels (c) and (d) show the mean velocities of 190 nm PTB7 nanoparticles along the x and y directions, recorded under chopped 980 nm illumination of ratchets. In (c), the time that the optical potential remained off (τ_{off}) was varied, with the intensity of 980 nm illumination kept constant at ~ 0.67 kW.cm^{-2}. In (d), the intensity of the 980 nm illumination was varied, whist τ_{off} was kept constant at its optimum value of 27.8 ms. The shaded region in (c) represents the theoretically-predicted range of τ_{off} values where ratcheting should be possible.

ing high-power operation, there were no observations of sudden ejection of particles from the illuminated area, abrupt large lateral displacements, or the appearance of new bright scattering features that would indicate bubble nucleation.

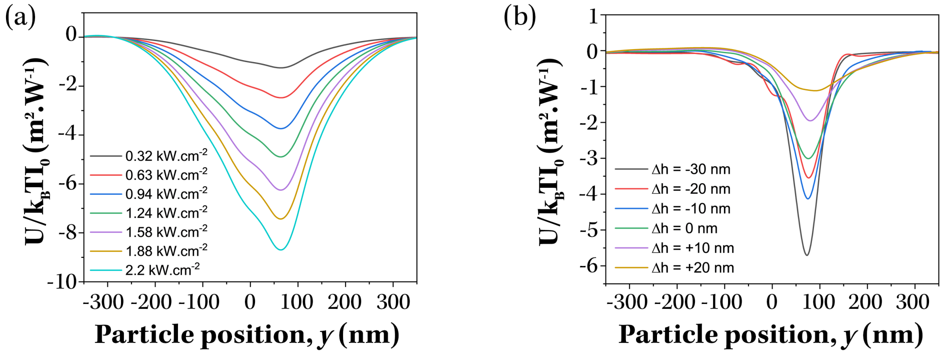

Figure S10: Numerical simulations of potentials for 40 nm PS spheres above plasmonic ratchets (a) operated at different incident intensities; and (b) at different heights above the ratchet. Potentials were calculated using the dipolar approximation. In (b), $\Delta h = 0$ nm corresponds to 10 nm separation between bottom of PS sphere and top surface of plasmonic ratchet, which is the separation used in the MST calculations described above. Results shown in panel (a) were calculated for $\Delta h = 0$ nm.

To determine whether nonlinear optical effects or changes in field distribution could have been responsible for altering the trapping potential at higher powers, numerical simulations were carried out for a range of excitation intensities (Figure S10(a)). The results showed that the optical potential was expected to scale linearly with incident power, with no change in its profile. This behavior indicates that the system operates within the linear response regime of the plasmonic structures, with no evidence of nonlinear field enhancement, thermal reshaping, or saturation effects. However, the experimentally measured trap stiffness increased much more rapidly - nearly exponentially - with power (Figure 5(c) of the main text), suggesting that additional interactions become significant at higher intensities.

Additional simulations performed for particles positioned at different heights above the antenna surface (Figure S10(b)) showed that the potential deepens as the particle approaches the surface, approximately doubling in depth 30 nm closer to the antenna, while also becoming more symmetric. Although this indicates stronger confinement close to the metal, the magnitude of the change is insufficient to explain the nearly exponential increase in experimentally measured trap stiffness with power. Taken together, the simulations indicate that purely optical effects cannot account for the rapid increase in stiffness observed experimentally, pointing instead to additional interactions arising at short particle–surface separations

under strong excitation.

Further evidence for additional near-surface interactions is provided by videos (available through Nano letters' website) of particle motion recorded at higher excitation powers. At incident intensities above approximately 0.8 kW cm^{-2}, a subset of particles appeared dimmer and nearly motionless, consistent with immobilization below the focal plane near the antenna tops. Other particles exhibited weak two-level fluctuations in intensity, suggestive of intermittent detachment and reattachment due to a reversible contact with the surface. These observations support the hypothesis that transient adhesion occurs between the particles and the antenna surfaces under strong optical excitation. Because neither the PS spheres nor the gold antennas were surface-functionalized, this adhesion was likely mediated by van der Waals forces that became significant once the optical gradient force drove particles into nanometre-scale proximity with the metal. During the potential-off phase of the modulation cycle, such particles would then need to first overcome these adhesive interactions before diffusing laterally, which effectively shortened their free-diffusion period and diminished the rectification efficiency of our plasmonic ratchets.

References

(1) Johnson, P. B.; Christy, R. W. Optical Constants of the Noble Metals. *Phys. Rev. B* **1972**, *6*, 4370–4379.

(2) Johnson, P. B.; Christy, R. W. Optical constants of transition metals: Ti, V, Cr, Mn, Fe, Co, Ni, and Pd. *Phys. Rev. B* **1974**, *9*, 5056–5070.

(3) Novotny, L.; Hecht, B. *Principles of Nano-Optics*; Cambridge University Press: Cambridge, UK, 2006.

(4) Griffiths, D. J. *Introduction to electrodynamics*; Pearson, 2013.

(5) Nina G. Sultanova, I. N., S. Kasarova Dispersion Properties of Optical Polymers. *Acta Physica Polonica Series a 116*.

(6) Stelling, C.; Singh, C. R.; Karg, M.; K onig, T. A. F.; Thelakkat, M.; Retsch, M. Plasmonic nanomeshes: their ambivalent role as transparent electrodes in organic solar cells. *Sci. Rep.* **2017**, *7*, 42530.

(7) aterston J. J.; William, S. J. I. On the physics of media that are composed of free and perfectly elastic molecules in a state of motion. *Philosophical Transactions of the Royal Society of London A* **1892**, 1831–1879.

(8) Boltzmann, L. *Vorlesungen über Gastheorie: Th. Theorie van der Waals'; Gase mit zusammengesetzten Molekülen; Gasdissociation*; Schlussbemerkungen: Leipzig, 1898.

(9) Zheng, F. Thermophoresis of spherical and non-spherical particles: a review of theories and experiments. *Advances in Colloid and Interface Science* **2002**, *97*, 255–28.

(10) Baffou, G.; Quidant, R. Thermo-plasmonics: using metallic nanostructures as nano-sources of heat. *Laser & Photonics Reviews* **2013**, *7*, 171–187.

(11) Wang, K.; Crozier, K. B. Plasmonic Trapping with a Gold Nanopillar. *ChemPhysChem* **2012**, *13*, 2639–2648.

(12) Jones, S.; Andrén, D.; Karpinski, P.; Käll, M. Photothermal Heating of Plasmonic Nanoantennas: Influence on Trapped Particle Dynamics and Colloid Distribution. *ACS Photonics* **2018**, *5*, 2878–2887.

(13) Boulais, E.; Lachaine, R.; Hatef, A.; Meunier, M. Plasmonics for pulsed-laser cell nanosurgery: Fundamentals and applications. *Journal of Photochemistry and Photobiology C: Photochemistry Reviews* **2013**, *17*, 26–49.
